# Nationwide utilization patterns and perioperative outcomes of minimally invasive pediatric nephrectomy in Germany

**DOI:** 10.3389/fped.2026.1851883

**Published:** 2026-08-25

**Authors:** Johannes Weidner, Jochen Blaser, Adan Chari Jirmo, Lucas Wennemann, Mikal Obed, Sören Wiesner, Jan Zeidler, Alejandro Daniel Hofmann, Jens Dingemann, Nagoud Schukfeh

**Affiliations:** 1 Department of Pediatric and Adolescent Surgery, Hannover Medical School, Hannover, Germany; 2 Representative Office of Lower Saxony, Techniker Krankenkasse (Health Insurance), Hannover, Lower Saxony, Germany; 3 Institute of Biostatistics, Hannover Medical School, Hannover, Germany; 4 Center for Health Economics Research Hannover (CHERH), Leibniz University Hannover, Hannover, Germany

**Keywords:** children, costs, health insurance, laparoscopy, renal tumor, retroperitoneoscopy

## Abstract

**Introduction:**

Minimally invasive nephrectomy is the favored approach for pediatric nephrectomies (74%) according to recent literature. However, data on treatment costs when compared to open nephrectomy are lacking. Our aim was to investigate the rate of minimally invasive pediatric nephrectomies in Germany and to assess treatment costs depending on different approaches.

**Materials and methods:**

Our study was based on data of the German statutory health care insurance company Techniker Krankenkasse from 01/2019-05/2024. After ethical approval, data of all patients up to the age of 18 years who underwent nephrectomy were analyzed with focus on surgical approach, malignancy, complications, ventilation hours, length of hospital stay and treatment costs. Database was queried for specific ICD-10-GM (International Statistical Classification of Diseases and Related Health Problems, 10th revision, German Modification) and OPS (Operationen- und Prozedurenschlüssel) coding. To enable international comparison, a literature search was conducted.

**Results:**

209 nephrectomies were performed in 209 patients during the study period. Out of these, 32% were minimally invasive. Postoperative blood transfusions (1 vs. 39) and readmissions within 90 days after discharge (18 vs. 84) were lower after minimally invasive nephrectomy. Postoperative ventilation hours were significantly lower after minimally invasive compared to open nephrectomies (0 vs. 5.9 ± 24.4 h, *p* < 0.0175). Minimally invasive nephrectomies were associated with almost half the length stay (7.9 ± 7 vs. 14.2 ± 21.7days, *p* < 0.0001) and half the treatment costs (10,782 ± 3,924€ vs. 21,240 ± 29,407€, *p* < 0.0001).

**Conclusion:**

In this nationwide administrative database, minimally invasive nephrectomy accounted for approximately one-third of pediatric nephrectomies. Descriptively, shorter hospital stays and lower hospital costs were recorded among patients undergoing minimally invasive nephrectomy. Given the descriptive nature of this analysis and the limited clinical detail available in the administrative database, these observations should not be interpreted as evidence of comparative effectiveness or causality and may, in part, reflect differences in case selection.

## 1 Introduction

Minimally invasive surgery has become standard and is now utilized in an increasing number of pediatric surgery departments worldwide. Currently, minimally invasive surgery is conducted in up to 80% of all abdominal pediatric surgical procedures (1). Minimally invasive nephrectomy is performed by transabdominal or retroperitoneal approach. Both approaches yield comparable safety and efficacy (2, 3). Data on rates of laparoscopic nephrectomy in children collected between 1998 and 2010 showed an increase in utilization of minimally invasive techniques from 1% to 12% during the study period (4). Recently, rates of up to 74% have been reported in a United Kingdom cohort (5). Also, tumor nephrectomy, representing a possibly challenging subgroup of operations, have been performed laparoscopically in almost half of the patients in a recent series also from the United Kingdom (6). Advantages of minimally invasive nephrectomy are lower rates of complications, shorter length of hospital stay, and decreased postoperative pain medication (7, 8).

While economic considerations are becoming increasingly important, comparative data on costs of minimally invasive vs. open nephrectomy, particularly on a population level, are scarce. In adults minimally invasive nephrectomy has been shown to be costs effective compared to open nephrectomy (9). To our knowledge, there is no data comparing treatment costs of minimally invasive vs. open nephrectomy in children.

Our aim was to investigate the rate of minimally invasive nephrectomy in Germany including data on complications, postoperative ventilation hours, length of hospital stay and treatment costs. We hypothesized that minimally invasive nephrectomy in children is still underutilized and associated with lower treatment costs compared to the open approach.

## 2 Methods

This cross-sectional study was based on data of the German statutory health insurance company Techniker Krankenkasse, which covers about 13% of the German population (12 million clients) (10). Data of all pediatric clients up to the age of 18 years who underwent nephrectomy from 01/2019 until 05/2024 and who had been continuous members of the Techniker Krankenkasse were analyzed. Patient-specific diagnoses were encoded based on the German Modification of the International Classification of Diseases in its 10th version (ICD-10-GM) and surgical procedures were encoded based on the Operationen- und Prozedurenschlüssel (OPS). The database provided pseudonymized information on type of treating institution, patient characteristics, operative data, and perioperative complications. Nephrectomy was encoded with 5-554.x and following subgroups according to OPS. Malignant neoplasm of the kidney was encoded as C64. We defined perioperative complications as postoperative bleeding (T81.0), blood transfusion (8–800), wound dehiscence (T81.3), infection (T81.4), sepsis (A41.-) and paralytic ileus (K56.0) according to ICD-10-GM and OPS-coding. Readmissions within 90 days after discharge have been queried. Statistical analyses were performed using GraphPad Prism version 9.5.1. The Mann–Whitney U test was used for comparisons between two groups, while the Kruskal–Wallis test followed by Dunn’s multiple comparisons test was applied for comparisons involving more than two groups. A two-tailed *p*-value of less than 0.05 was considered statistically significant. Unless stated otherwise, data are given as mean ± standard deviation. Reported *p*-values and significances are interpreted exploratory. A literature search according to PRISMA guidelines was carried out to enable an international comparison using the query [(pediatric) AND (nephrectomy)] AND (laparoscopy)) OR (retroperitoneoscopic). Manuscripts published between 2010 and 2025 were identified from PubMed database (https://www.pubmed.com). Ethical approval was obtained by the local ethics committee of Hannover Medical School (11391_BO_K_2024).

## 3 Results

209 patients aged 0–18 years (mean 5.5 ± 5.3 years) who underwent a total of 209 nephrectomies were identified. Out of these, 66 (32%) were encoded as minimally invasive nephrectomy. 57 (27%) tumor nephrectomies were carried out in patients diagnosed with malignant neoplasm of the kidney. No minimally invasive tumor nephrectomy was identified. When tumor nephrectomies were excluded, the rate of minimally invasive nephrectomy was 43% (Table 1). No differences in surgical approach were observed according to the type of treating institution.

**Table 1:** Surgical procedure depending on entity. Number of procedures (%). .

Entity	Minimally invasive nephrectomy	Open nephrectomy
Non-tumor	66 (43%)	86 (57%)
Tumor	0 (0%)	57 (%)
Total	66 (32%)	143 (68%)

**Figure 1:**
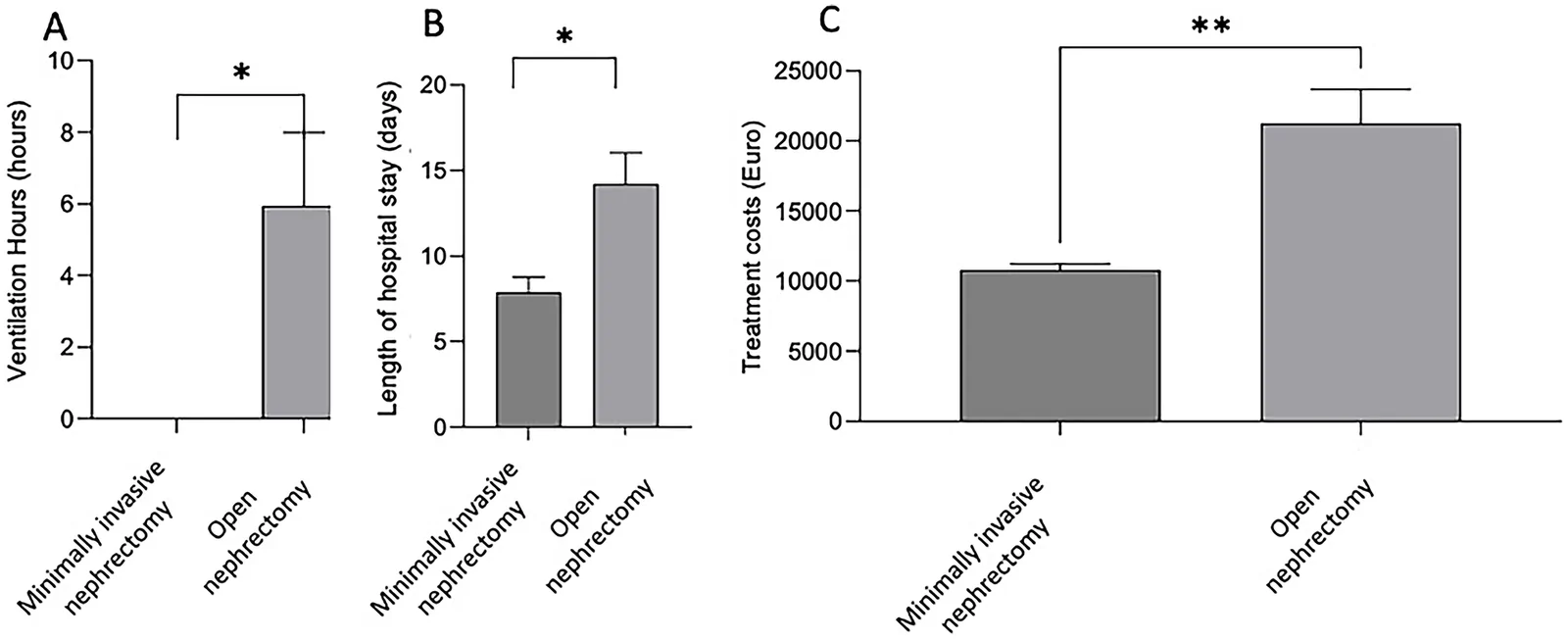
Postoperative outcomes after minimally invasive and open nephrectomy (minimally invasive nephrectomy: *n* = 66; open nephrectomy: *n* = 143). **A** Mean postoperative ventilation time (hours) by surgical approach. **B** Mean length of hospital stay (days) by surgical approach. **C** Mean treatment costs by surgical approach. Statistical analyses were performed using the non-parametric Mann–Whitney test, and exact two-tailed *p* values are indicated in the graphs.

Regarding complications, postoperative bleeding was encoded in 1 case of minimally invasive (1.5%) and in 7 cases of open nephrectomy (4.9%). Blood transfusion was encoded in 1 (1.5%) case after minimally invasive and 39 (27.3%) cases after open nephrectomy. No cases of infection or sepsis were recorded in the minimally invasive group, and 2 cases of sepsis occurred the open nephrectomy group (1.4%). There was no paralytic ileus encoded in the minimally invasive and 2 cases in the open nephrectomy group (1.4%). Mortality was 0% in the minimally invasive and 0.7% (*n* = 1) in the open nephrectomy group (Table 2). According to the encoded diagnoses this patient died of multi organ failure post aspiration. None of the patients in the minimally invasive group required postoperative ventilation, patients in the open nephrectomy group required postoperative ventilation with a mean duration of 5.9 ± 24.4 h (*p* < 0.0175, Figure 1A).

**Table 2:** Surgical complications according to surgical approach and entity. Number of complications (%; 95% confidence interval). .

Complication	Minimally invasive	Open – non tumor	Open – tumor
Bleeding	1 (1.5%; 0.3%–8.1%)	3 (3.5%; 1.2%–9.8%)	4 (7.0%; 2.8–16.7%)
Blood transfusion	1 (1.5%; 0.3%–8.1%)	16 (18.6%; 11.8–28.1%)	23 (40.4%; 28.6–53.3%)
Wound dehiscence	0 (0%; 0%–5.5%)	0 (0%; 0%–4.3%)	0 (0%; 0%–6.3%)
Infection	0 (0%; 0%–5.5%)	0 (0%; 0%–4.3%)	0 (0%; 0%–6.3%)
Sepsis	0 (0%; 0%–5.5%)	2 (2.3%; 0.6%–8.1%)	0 (0%; 0%–6.3%)
Paralytic ileus	0 (0%; 0%–5.5%)	2 (2.3%; 0.6%–8.1%)	0 (0%; 0%–6.3%)
In-hospital death	0 (0%; 0%–5.5%)	1 (1.2%; 0.2%–6.3%)	0 (0%; 0%–6.3%)
Reoperations	0 (0%; 0%–5.5%)	4 (4.7%; 1.8–11.4%)	1 (1.8%; 0.3%–9.3%)
Readmission	18 (27.3%; 18.0–39.0%)	39 (45.3%; 35.3–55.8%)	45 (78.9%; 66.7–87.5%)

Mean length of hospital stay was 7.9 ± 7 days in the minimally invasive group and 14.2 ± 21.7 in the open group (*p* < 0.0001) (Figure 1B).

Within 90 days after discharge there were no reoperations after minimally invasive and 5 (3.5%) after open nephrectomy. Readmission within 90 days of surgery occurred in 18 (27.3%) cases after minimally invasive and 84 (58.7%) cases after open nephrectomy. For subgroup analysis excluding tumor nephrectomy see Table 2.

Figure 1C: Treatment costs for the corresponding hospital stay depending on surgical approach. The mean costs for minimally invasive nephrectomy were only half those of the open approach (10,782 ± 3,924 € vs. 21,240 ± 29,407€, *p* < 0.0001).

**Figure 2:**
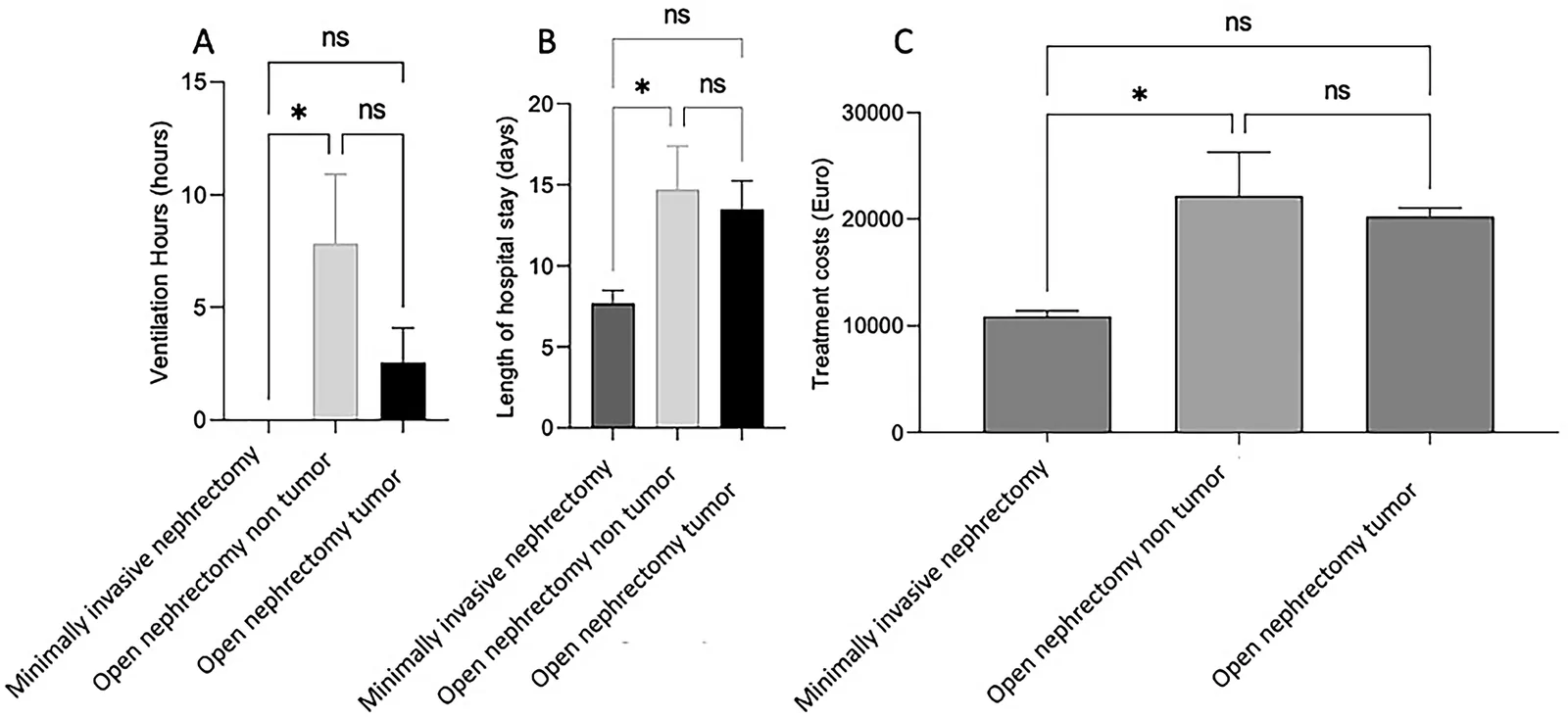
Subgroup analyses of postoperative outcomes between minimally invasive and open nephrectomy (minimally invasive nephrectomy: *n* = 66; open nephrectomy non-tumor: = 86, open nephrectomy tumor: *n* = 57). **A** Mean postoperative ventilation time (hours) by surgical approach and entity. **B** Mean length of hospital stay (days) by surgical approach and entity. **C** Mean treatment costs by surgical approach and entity. Statistical analyses were performed using the non-parametric Kruskal–Wallis test followed by Dunn's multiple comparisons test. Exact *p* values are indicated in the graphs.

In order to reduce potential bias, a subgroup analysis was performed comparing minimally invasive, open non-tumor and open tumor nephrectomy. Results can be seen in Figure 2.

**Figure 3:**
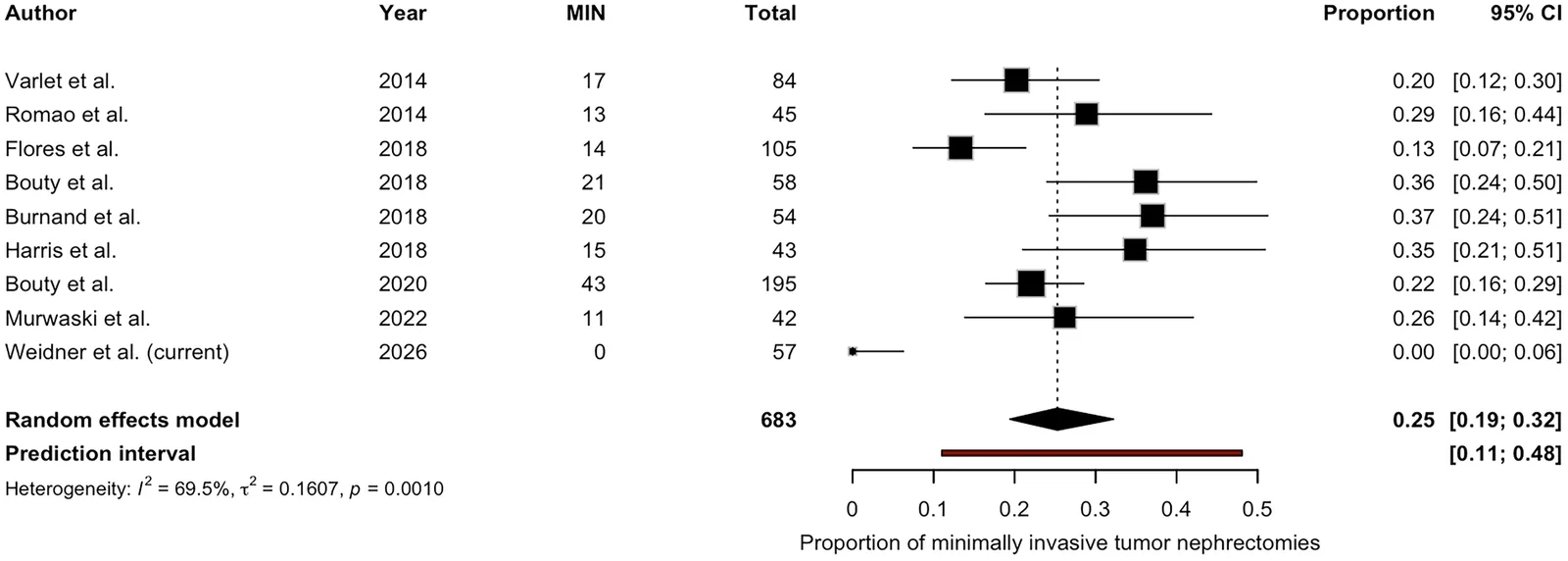
Forest plot showing the proportion of minimally invasive tumor nephrectomies across the included studies and the current cohort. Study-specific proportions with 95% confidence intervals are presented together with the pooled estimate from the random-effects model and the corresponding prediction interval.

**Figure 4:**
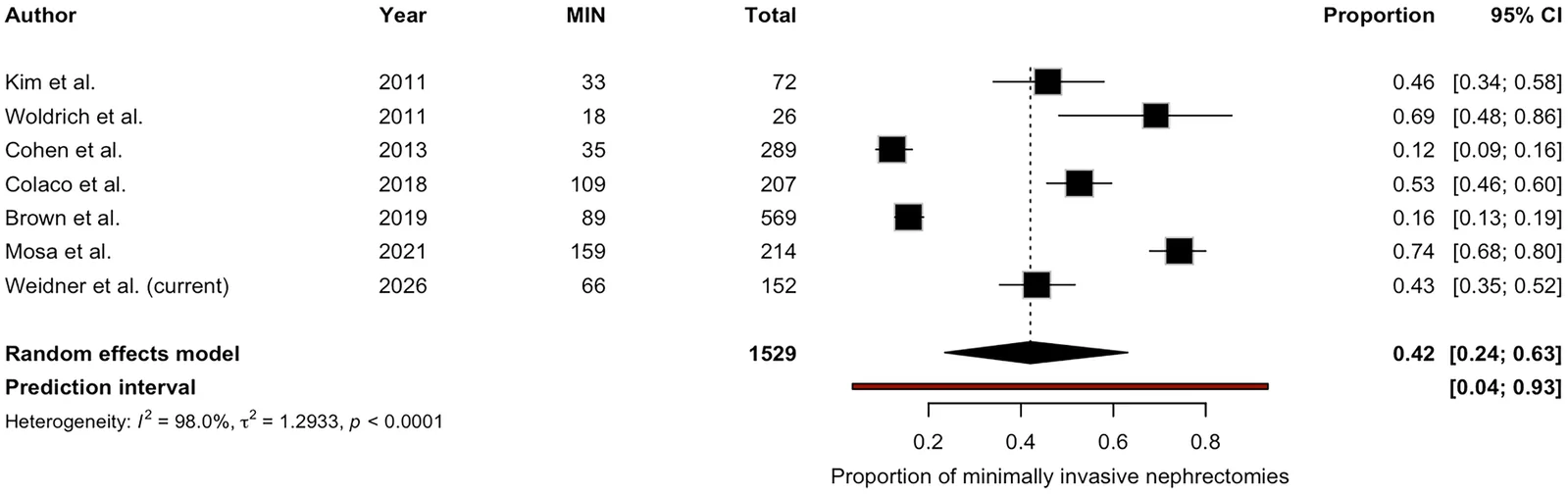
Forest plot showing the proportion of minimally invasive nephrectomies across the included studies and the current cohort. Study-specific proportions with 95% confidence intervals are presented together with the pooled estimate from the random-effects model and the corresponding prediction interval.

To place our data in the context of the current international literature and to provide an international comparison, we conducted a literature search according to PRISMA guidelines and obtained the results shown in Tables 3, 4 as well as Figures 3, 4. Details on the review process including references are Supplementary Material 1.

**Table 3:** Reports including more than 10 cases of pediatric tumor nephrectomies. *n* = number, MIN = minimally invasive nephrectomy, ON = open nephrectomy.

Author, Year	Country	Study period	Study type	n	MIN	ON	Reported parameter
Murawski et al., 2022 (31)	Poland	2013-2020	Retrospective, single center	42	11 (26%)	31 (74%)	Tumor volume, length of operation, postoperative complication, length of hospital stay
Bouty et al., 2020 (32)	France, Australia	2006-2018	Retrospective, multi center	195	43 (22%)	152 (78%)	Imaging, surgical procedure, complications, follow up
Flores et al., 2018 (33)	Argentina	2010-2016	Retrospective, single center	105	14 (13%)	91 (87%)	Imaging, survival, relapse
Bouty et al., 2018 (34)	Australia	2010-2017	Retrospective, single center	58	21 (41%)	37 (59%)	Tumor volume, complications, relapse
Burnand et al., 2018 (35)	Australia	2010-2017	Retrospective, single center	54	20 (37%)	34 (63%)	Imaging, complications, oncologic outcome
Harris et al., 2018 (36)	UK	2002-2016	Retrospective, single center	43	15 (35%)	28 (65%)	Tumor volume, complications
Varlet et al., 2014 (37)	France	2005-2012	Retrospective, multi center	84	17 (20%)	67 (80%)	Complications, tumor size, follow-up
Romao et al., 2014 (38)	Canada	2006-2001	Retrospective, single center	45	13 (29%)	32 (71%)	Tumor rupture, length of stay, analgesia
Weidner et al. (current study), 2026	Germany	2020-2024	Retrospective, population based	57	0 (0%)	57 (100%)	Complications, ventilation hours, length of hospital stay, costs

**Table 4:** Reports including more than 10 cases of pediatric non-tumor nephrectomies. N, number; MIN, minimally invasive nephrectomy; ON, open nephrectomy.

Author, Year	Country	Study period	Study type	n	MIN	ON	Reported parameter
Mosa et al., 2021 (5)	UK	2009-2020	Retrospective, single center	214	159 (74%)	54 (26%)	Operative time, hospital stay, complications, conversions
Brown et al., 2019 (39)	USA	2006-2015	Retrospective, population based	569	89 (16%)	479 (84%)	Multicystic dysplastic kidney, annual trends
Colaco et al., 2018 (40)	USA	2014	Retrospective, population based	207	109 (53%)	98 (47%)	Length of stay, complications
Cohen et al., 2013 (41)	USA	2000-2011	Retrospective, population based	289	35 (12%)	254 88%)	Length of stay, ICU-admission, mortality, readmission
Kim et al., 2011 (8)	USA	2006-2009	Retrospective, single center	72	33 (46%)	39 (54%)	Operative time, analgesia, length of stay
Woldrich et al., 2011 (42)	USA	2007-2011	Retrospective, single center	26	18 (69%)	8 (31%)	Operative time, blood loss, complications
Weidner et al. (current study) 2026	Germany	2020-2024	Retrospective, population based	152	66 (43%)	86 (57%)	Complications, ventilation hours, length of hospital stay, costs

## 4 Discussion

Over the past decades, minimally invasive nephrectomy has been increasingly adopted in clinical practice. Nowadays, this approach is regarded the standard procedure in adults (11). Recently, Mosa et al. published a single center study from the United Kingdom reporting on 74% of minimally invasive nephrectomies in pediatric patients (5). In this nationwide administrative database, 32% of nephrectomies were encoded as minimally invasive procedures. This proportion should be interpreted as a descriptive finding based on administrative coding data.

Several studies have compared complication rates between minimally invasive and open nephrectomy. Previously, our group analyzed 43 meta-analyses drawing the conclusion that lower complication rates are a typical advantage of minimally invasive surgery (1). Sarhan reported on an overall complication rate for minimally invasive nephrectomy of 22% for the transperitoneal and 13% for the retroperitoneal approach in a pediatric cohort (2). Furthermore, Nazzani et al. published wound complication rates of 1% after minimally invasive and 3% after open nephrectomy in adults. Similarly, significantly lower complication rates after minimally invasive compared to open nephrectomy (19% vs. 23%) have been observed in an adult population by Franco et al. (12). In our nationwide administrative database, postoperative complication frequencies were described for minimally invasive and open nephrectomy. Postoperative blood transfusions and readmissions were recorded in a smaller proportion of patients undergoing minimally invasive nephrectomy. As this study is purely descriptive, these observations should not be interpreted as comparative evidence of differences in surgical outcomes. Direct comparison with previously published complication rates remains challenging because of differences in study populations, data sources, and complication definitions.

Data on ventilation time after nephrectomy is not available in the existing literature. There was no need for postoperative ventilation after minimally invasive nephrectomy. After open nephrectomy a mean of 7.6 h has been encoded. Comparable findings were reported by Gil et al., revealing a one-day reduction in ventilation time following minimally invasive repair of congenital anomalies in children (13). While these findings may provide context for our observations, they cannot be directly extrapolated to pediatric nephrectomy. In general, evidence from pediatric surgical populations suggests that prolonged postoperative mechanical ventilation is associated with increased healthcare resource utilization, including longer hospital stays and higher costs. Although these findings originate from different surgical settings, they indicate that prolonged ventilatory support may contribute to increased postoperative resource use (14, 15).

Shorter length of hospital stay has been observed before after minimally invasive compared to open nephrectomy in pediatric patients (8). These finding are underlined by above-mentioned analysis of meta-analyses concerning minimally invasive pediatric surgery conducted by our department (1). Consistent with prior studies, our results show that minimally invasive nephrectomy is associated with a significantly shorter hospital stay compared to the open approach.

Amongst others operative indications for nephrectomy include vesicoureteral reflux, ectopic ureter, ureteropelvic junction obstruction, and multicystic dysplastic kidney. In some cases, nephrectomy is indicated due to renal neoplasms. 90% of malignant renal tumors in children are nephroblastoma (16). According to the International Society of Pediatric Oncology (SIOP) guidelines, primary resection is indicated in case of nephroblastoma in Europe (17). Concerning the operative approach, SIOP clinical guidelines for the management of children with nephroblastoma recommend the open approach (18). However, under certain prerequisites like small central tumors or absence of thrombus in the renal vein, laparoscopic nephrectomy is possible (18). Similarly, the National Comprehensive Cancer Network (NCCN) guidelines do not explicitly state whether to use minimally invasive or open nephrectomy (16). A meta-analysis published in 2024 by Mentessidou et al. including 7 studies on pediatric renal tumors and 5 studies on nephroblastoma revealed that minimally invasive nephrectomy is a feasible option for selected post chemotherapy small central renal tumors in children. In this meta-analysis 122 minimally invasive and 210 open tumor nephrectomies have been compared without differences in intraoperative or postoperative complications. The only aspect that appeared to be inferior in minimally invasive tumor nephrectomy was the number of lymph nodes taken as samples (6). In contrast, a meta-analysis published in 2025 by Lacerda et al. reported no statistically significant differences in lymph node harvest, and disease or local recurrence (19). Given these conflicting results, further research to elucidate possibilities for minimally invasive tumor nephrectomy is mandatory. In our study, all tumors were resected via an open approach. While minimally invasive nephrectomy may be appropriate in selected oncological settings, the lack of detailed clinical and oncological information precludes any conclusions regarding the appropriateness of the surgical approach in our cohort.

Economic considerations have been gaining increased importance in the recent years (20). Several studies have shown lower costs for laparoscopic procedures. Evidence from adult populations reported by Lotan et al. and Buse et al. demonstrates that minimally invasive nephrectomy is cost-effective compared with the open approach (9, 21). In a pediatric population, laparoscopic Nissen fundoplication has been shown to be cost effective compared to the open approach (22). In contrast, minimally invasive and open esophageal atresia repair has been shown to have comparable treatment costs (23). To our knowledge, our study provides data on medical treatment costs of minimal invasive vs. open nephrectomies in a pediatric population for the first time.

A known disadvantage of minimally invasive procedures, however, is higher costs which, in most cases are eliminated by shorter length of hospital stay as has been shown by Lotan et al. (9). Additionally, Mahomed et al. published data on effective cost-saving strategies, like standardization of operating packs and the use of reusables, for laparoscopic procedures in pediatric settings without compromising on patient safety (24).

One aspect possibly hampering a broad implementation of a technically demanding minimally invasive nephrectomy is the combination of low case volume in pediatric surgery and achieving a reasonable learning curve (25). In adult populations variable numbers of 30–200 minimally invasive procedures have been proposed to reach stable conversion rates and peri- and postoperative outcomes (26–28). To overcome this considerably steep learning curve several training models have been proposed.

Zahradníková et al. analyzed 72 studies on simulation models, five of which focused on urology, and recommended structured pediatric surgical education using validated models (29). In accordance, Davidson et al. reported on different training models in fetal and neonatal surgery highlighting the importance of animal models providing the opportunity to evaluate complications and long-term outcomes (30).

Our study has limitations. As this was an exploratory study, we did not conduct a power calculation. Accordingly, both non-significant and non-significant results should be interpreted with caution. Additionally, a coding bias cannot be ruled out. Furthermore, the ICD-10-GM code C64 does not differentiate between tumor subtypes or stages of severity. The preferential selection of less complex cases for minimally invasive nephrectomy and the resulting selection bias cannot be excluded. Owing to the lack of detailed clinical information in the administrative database, residual confounding may have influenced the observed differences between the study groups. Observed differences should be interpreted as associations rather than causal effects of the surgical approach.

## 5 Conclusion

In this nationwide administrative database, minimally invasive nephrectomy accounted for approximately one-third of pediatric nephrectomies. Descriptively, shorter hospital stays and lower hospital costs were recorded among patients undergoing minimally invasive nephrectomy. Given the descriptive nature of this analysis and the limited clinical detail available in the administrative database, these observations should not be interpreted as evidence of comparative effectiveness or causality and may, in part, reflect differences in case selection. Prospective studies with detailed clinical data are needed to better define indications for minimally invasive nephrectomy in children and to further evaluate perioperative and long-term outcomes.

## Data Availability

The raw data supporting the conclusions of this article will be made available by the authors, without undue reservation.
