# Supplementary material for: Nationwide utilization patterns and perioperative outcomes of minimally invasive pediatric nephrectomy in Germany
Source: Front Pediatr. 2026 Aug 25;14:1851883. doi: 10.3389/fped.2026.1851883 (PMC13550151; doi:10.3389/fped.2026.1851883)
Supplement: Supplementary file 1 [file Table1.docx]

**((pediatric) AND (nephrectomy)) AND (laparoscopy)) OR (retroperitoneoscopic)**

**Identification of studies via databases and registers**

Records identified from pubmed (n = 1,027)

**Identification**

Records screened

(n = 1,027)

Records excluded

(n = 0)

Reports sought for retrieval

(n = 1,027)

Reports not retrieved

(n = 983)

**Screening**

Reports assessed for eligibility

(n = 55)

Reports excluded: (n = 41)

no open controls (n = 31)

n < 10 (n = 7)

no full text (n = 2)

meta-analysis (n = 1)

Studies included in review

(n = 14)

**Included**

Source: Page MJ, et al. BMJ 2021;372:n71. doi: 10.1136/bmj.n71.

This work is licensed under CC BY 4.0. To view a copy of this license, visit <https://creativecommons.org/licenses/by/4.0/>
